# Analysis of parameters in critically ill patients influencing amikacin peak levels

**DOI:** 10.1186/cc12020

**Published:** 2013-03-19

**Authors:** IM Hollevoet, SJ Toye, J De Waele

**Affiliations:** 1 UZ Gent, Belgium; 2 Stijn Toye, Gent, Belgium

## Introduction

Recent studies demonstrate that a loading dose of 25 mg/kg (total body weight) of amikacin in septic patients is required to reach a sufficient peak concentration. This study examines parameters influencing the relation between amikacin dose and peak concentration.

## Methods

In this retrospective study we looked at 47 patients (128 peak levels) between 2003 and 2012. Multivariate linear regression analysis was done for several parameters: administered dose calculated with total body weight, ideal body weight, adjusted body weight, type of intensive care patient, BMI, daily fluid balance, SOFA score and APACHE score, and patient characteristics were analyzed.

## Results

**Figure 1:**
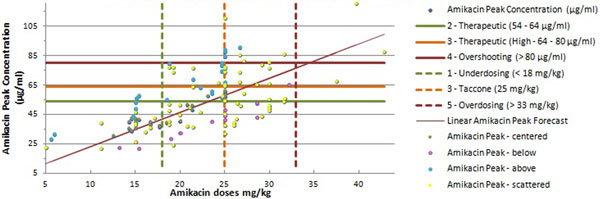
**Correlation between dose of amikacin and peak concentration**.

A linear correlation between dose and amikacin peak level was confirmed (Figure 1). A total 54.69% of all amikacin administrations did not result in a therapeutic peak level. The multivariate linear regression analysis showed the best linear correlation with adjusted body weight and SOFA score. The comparison of variables between four patient groups, based on the deviation between measured peak level and predicted peak level (according the linear correlation), showed new variables that may influence peak level.

## Conclusion

This confirms that low doses (<18 mg/kg) of amikacin in intensive care patients seldom result in a therapeutic peak level. The proposed loading dose of 25 mg/kg is good for reaching a therapeutic level, although 29.6% remains subtherapeutic. Due to the linear correlation, more therapeutic levels may be reached with higher doses (25 to 30 mg/kg). New variables need further investigation to explain the high variability in achieved peak level.
